# Association Between Purchase of Over-the-Counter Medications and Ovarian Cancer Diagnosis in the Cancer Loyalty Card Study (CLOCS): Observational Case-Control Study

**DOI:** 10.2196/41762

**Published:** 2023-01-26

**Authors:** Hannah R Brewer, Yasemin Hirst, Marc Chadeau-Hyam, Eric Johnson, Sudha Sundar, James M Flanagan

**Affiliations:** 1 Department of Surgery and Cancer Faculty of Medicine Imperial College London London United Kingdom; 2 Department of Behavioural Science and Health University College London London United Kingdom; 3 School of Public Health Faculty of Medicine Imperial College London London United Kingdom; 4 Institute of Cancer and Genomic Sciences University of Birmingham Birmingham United Kingdom

**Keywords:** ovarian cancer, early diagnosis, transactional data, health informatics, cancer risk, medication, self-medication, self-care, over-the-counter medication, nonspecific symptoms, pain medication, indigestion medication

## Abstract

**Background:**

Over-the-counter (OTC) medications are frequently used to self-care for nonspecific ovarian cancer symptoms prior to diagnosis. Monitoring such purchases may provide an opportunity for earlier diagnosis.

**Objective:**

The aim of the Cancer Loyalty Card Study (CLOCS) was to investigate purchases of OTC pain and indigestion medications prior to ovarian cancer diagnosis in women with and without ovarian cancer in the United Kingdom using loyalty card data.

**Methods:**

An observational case-control study was performed comparing purchases of OTC pain and indigestion medications prior to diagnosis in women with (n=153) and without (n=120) ovarian cancer using loyalty card data from two UK-based high street retailers. Monthly purchases of pain and indigestion medications for cases and controls were compared using the Fisher exact test, conditional logistic regression, and receiver operating characteristic (ROC) curve analysis.

**Results:**

Pain and indigestion medication purchases were increased among cases 8 months before diagnosis, with maximum discrimination between cases and controls 8 months before diagnosis (Fisher exact odds ratio [OR] 2.9, 95% CI 2.1-4.1). An increase in indigestion medication purchases was detected up to 9 months before diagnosis (adjusted conditional logistic regression OR 1.38, 95% CI 1.04-1.83). The ROC analysis for indigestion medication purchases showed a maximum area under the curve (AUC) at 13 months before diagnosis (AUC=0.65, 95% CI 0.57-0.73), which further improved when stratified to late-stage ovarian cancer (AUC=0.68, 95% CI 0.59-0.78).

**Conclusions:**

There is a difference in purchases of pain and indigestion medications among women with and without ovarian cancer up to 8 months before diagnosis. Facilitating earlier presentation among those who self-care for symptoms using this novel data source could improve ovarian cancer patients’ options for treatment and improve survival.

**Trial Registration:**

ClinicalTrials.gov NCT03994653; https://clinicaltrials.gov/ct2/show/NCT03994653

## Introduction

Ovarian cancer is the eighth most common cancer in females worldwide and is the sixth most common cause of cancer-related death in females in the United Kingdom [1,2]. There is an urgent need for earlier diagnosis to improve survival outcomes since 5-year survival rates decrease from 93% for women diagnosed at the earliest stage to 20% for women diagnosed at the latest stage[2]; however, a population-based ovarian cancer screening program is not currently recommended in the United Kingdom [3]. One possible approach for improvement is to encourage earlier help-seeking behavior among women experiencing symptoms such as abdominal/pelvic pain, bloating, loss of appetite, and urinary symptoms, which can often be associated with other more common illnesses [4]. Awareness of these nonspecific symptoms among women in the United Kingdom is low [5,6]; consequently, the average time between experiencing a symptom and presenting to primary care with these symptoms is 39 days [7].

A proof-of-concept study conducted in 2019 concluded that there may be an increase in purchases of pain and indigestion medications 10-12 months before ovarian cancer diagnosis to treat symptoms [8], although larger studies were needed to validate this finding. The Cancer Loyalty Card Study (CLOCS) was therefore proposed to investigate this further through an observational case-control study design, which hypothesized that a significant change in purchasing behavior, specifically an increase in self-medication prior to presentation in primary care, could be an indication for early signs of ovarian cancer [9]. The primary objective of the CLOCS is to investigate the purchasing behaviors of women with and without ovarian cancer using commercial data collected through loyalty card use at two participating high street retailers in the United Kingdom with the aim to reduce delays in ovarian cancer diagnosis.

## Methods

### Study Design, Setting, and Participants

The CLOCS case-control study design and protocol have been described previously [9]. Eligible participants were women in the United Kingdom, 18 years or older, who owned at least one of the participating retailers’ loyalty cards. Patients diagnosed with ovarian cancer within 2 years of recruitment to the study until January 31, 2022, were considered cases, whereas controls did not have a previous diagnosis of ovarian cancer. When face-to-face interactions between patients with ovarian cancer and clinics were minimized due to the COVID-19 pandemic, the protocol was amended to allow for patients to be recruited from National Health Service (NHS) clinics over the phone.

**Figure 1:**
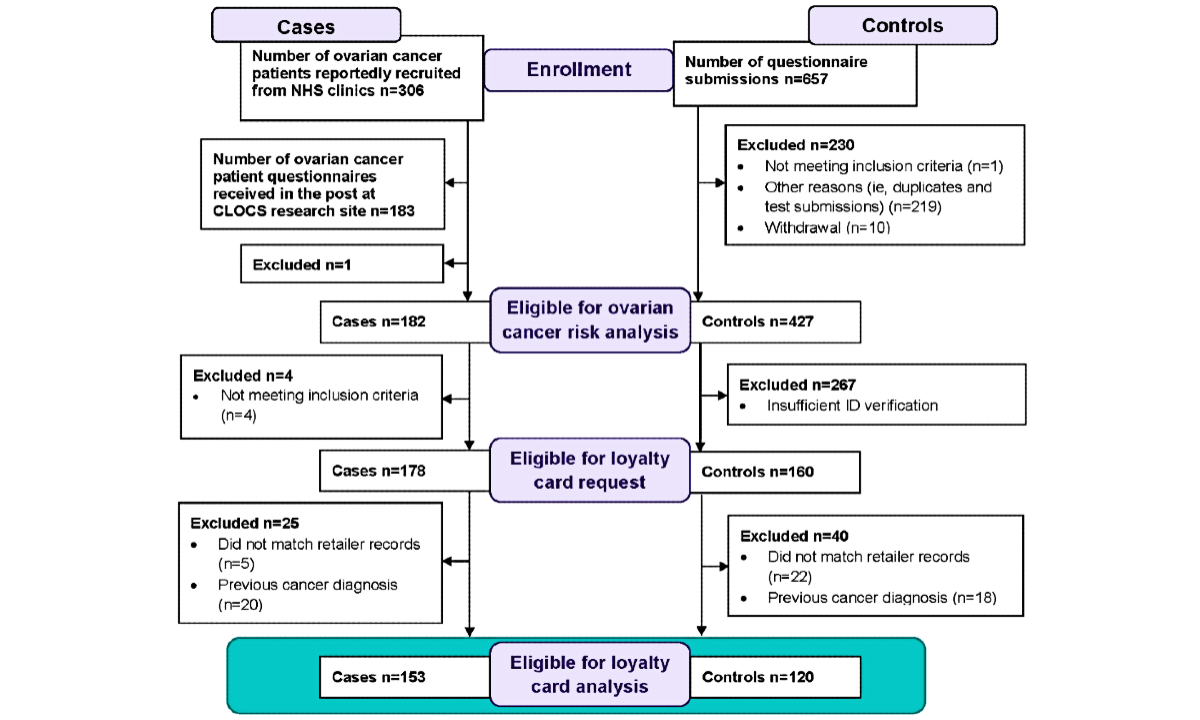
CONSORT (Consolidated Standards of Reporting Trials) diagram for Cancer Loyalty Card Study (CLOCS) case-control recruitment and loyalty card data collection. NHS: National Health Service.

Patients were recruited from 12 NHS clinic sites in England, Wales, and Scotland from November 1, 2019, to January 31, 2022. Participants without ovarian cancer were recruited indirectly through an online experiment that investigated the effects of an animated decision aid on willingness to take part in the CLOCS in July 2020 (unpublished), and directly through paid social media advertisements using Meta (formerly known as Facebook), a test recruitment email from one of the participating retailers, word of mouth, and the advocate network VOICE [10] from September 1, 2020, to May 31, 2021. A CONSORT (Consolidated Standards of Reporting Trials) diagram of CLOCS participant recruitment and eligibility for analysis is shown in Figure 1.

After obtaining explicit consent, up to 6 years of loyalty card purchase history, starting from the date of recruitment, was requested from two high street retailers, hereafter referred to as high street retailer 1 (HSR1) and high street retailer 2 (HSR2), for eligible participants using the name and number on their loyalty card(s). One of the participating retailers offers a supply of health and beauty items, while the other stocks a wider range of health, beauty, and grocery items, thereby providing CLOCS with a variety of purchases related to the participants’ intentions to shop at either retailer. If these details matched the retailers’ records, then the participant purchase history was transferred to the CLOCS team. If the details did not match, then the purchase history was not shared, and the participant was recontacted up to two times to clarify their details. Once clarified, the participant’s card history was requested once again as described previously [9].

### Patient and Public Involvement

The CLOCS team included two ovarian cancer patient advocates who were involved in the project from the inception of the idea to study design, ethical approval applications, and development of recruitment strategies throughout the CLOCS. We presented two public-facing seminars to engage with the wider audience about the CLOCS in December 2020 and March 2021 during Ovarian Cancer Awareness month. Furthermore, we held three annual scientific meetings inviting academics, patient representatives, and participating high street retailers.

### Variables

Participants completed a short questionnaire about ovarian cancer risk factors, including ethnicity, marital status, BMI, age at menarche, menopausal status, age at menopause, parity, breastfeeding, hysterectomy, tubal ligation, cancer history, endometriosis, aspirin use, oral contraceptive (OC) use, hormone replacement therapy (HRT) use, family history of ovarian and breast cancers, vaping, and cigarette smoking. The questionnaire also requested information about the symptoms experienced (if any) and number of visits to the general practitioner (GP) in the year before participating in the study for controls or leading up to cancer referral or diagnosis for cases.

In addition, participants were asked about which food and pharmacy stores they shop at regularly, at which stores they own a loyalty card, frequency of use of those loyalty cards, and where they heard about the study (for controls only). The questionnaire was amended to include a question about the number of people in their household. To gain a better understanding of the impact of COVID-19 on purchase history patterns and ovarian cancer diagnosis delays, the questionnaire was amended to include a question about whether participants had been diagnosed with COVID-19 (responses limited to the following: diagnosed and recovered, diagnosed and still ill, suspected but not formally diagnosed, did not have COVID-19). The date of diagnosis, type, stage, grade, *BRCA1* and *BRCA2* mutation status, and any surgical outcomes for cases were provided by the clinical team at their respective recruitment sites.

### Statistical Methods

#### Ovarian Cancer Risk

Unconditional logistic regression was used to calculate the risk of ovarian cancer among CLOCS participants, first using an unadjusted model and then with adjustment for age, OC use (ever/never), menopausal status (premenopausal/postmenopausal), and HRT use (ever/never).

An ovarian cancer risk score was calculated for all participants stratified by age (<50 years and ≥50 years) using an additive model described previously [11]. The risk score was the sum of the product of the median log odds ratio (OR) and the values of the following risk factors: low- and high-dose aspirin use (regular use ever/never), BMI (continuous), breastfeeding (ever/never), number of months breastfeeding (continuous), endometriosis (ever diagnosed/never), family history of breast cancer in first-degree relative (ever/never), family history of ovarian cancer in first-degree relative (ever/never), hysterectomy and no HRT use (ever/never), age at the end of the last pregnancy (continuous), age at menarche (continuous), menopausal status (pre-/postmenopausal), OC use (ever/never), duration of OC use (continuous), number of nonfull-term pregnancies (continuous), number of full-term pregnancies (continuous), and tubal ligation status (ever/never). Instead of limiting the analysis to participants with complete data or eliminate risk factors from the risk score, missing values were imputed using the k-nearest neighbors imputation method in the Visualization and Imputation of Missing Values (*vim*) package in R. Participants were grouped into low-, medium-, or high-risk score groups using cut-off points based on tertiles of the risk score among controls.

#### Purchase History Analysis

For purchase history analyses, two controls were matched with each case according to age (continuous) and which HSR loyalty card they had (HSR1 only, HSR2 only, HSR1 and HSR2), with replacement. The analysis was focused on the period of 24 months prior to diagnosis for cases to maximize the amount of purchase history data available from participants and to detect a difference in purchases between cases and controls up to 10 months before diagnosis, according to the prior power calculations [9]. Owing to the differing periods of recruitment of cases and control, where possible, the same 24 months of purchases from controls were aligned with each matched case leading up to diagnosis; otherwise, the latest 24 months of purchases were used for controls. Purchases of items commonly used to treat symptoms of ovarian cancer, such as stomach pain and indigestion, were averaged and cumulatively summed for each participant per month over 24 months. Pain and indigestion medication purchases were selected using the categories predetermined by the retailers.

For statistical analysis, purchases for each individual were summed on a sliding 3-month window to smooth the signal and to represent a pattern of individual purchase behavior for cases and controls comparing all purchases to the purchase of both pain and indigestion medication bought together. The Fisher exact test was used to calculate the ORs, 95% CIs, and significance of enrichment at each month prior to diagnosis.

A conditional logistic regression model was used to investigate the association between purchase of medications that treat pain and indigestion over the 24-month period as well as pain and indigestion medication purchases separately, where the medication purchase was the exposure and ovarian cancer diagnosis was the outcome.

A receiver operating characteristic (ROC) analysis was performed for purchases of pain and indigestion medications separately at each month prior to diagnosis for cases and the latest 24 months available for controls. For cases, the ROC analysis was repeated and stratified by stage at diagnosis, where stages 1 and 2 were grouped together and considered the early stage and stages 3 and 4 were considered the late stage.

Sensitivity analyses were performed to assess the impact of several factors on purchase behaviors among CLOCS participants. First, conditional logistic regression was used to assess the association between ovarian cancer diagnosis and purchases of pain and indigestion medications before and after the COVID-19 pandemic (before and after March 2020). Second, to account for seasonal purchase behaviors (eg, painkillers linked to flu and cold season in autumn and winter), the conditional logistic regression model was repeated with adjustment for seasons. Finally, to investigate whether the number of members in a household affects the association between ovarian cancer and purchases, the model was run with adjustment for the number of members in the household and OC use.

Further sensitivity analyses related to ovarian cancer symptom behaviors were performed using the same conditional logistic regression model with adjustment for whether participants reportedly attended the GP in the 12 months prior to diagnosis for cases and prior to participation for controls, as well as with adjustment for whether participants reported experiencing nonspecific ovarian cancer symptoms.

All statistical analyses were conducted in the ISO27001-certified Secure Enclave environment at Imperial College London using R (version 4.1.2).

### Ethics Considerations

The CLOCS was reviewed and approved by the North West-Greater Manchester South Research Ethics Committee (19/NW/0427). Explicit consent for the research team to request loyalty card data from participating retailers and to take part in the study was given from all participants.

## Results

### Participant Characteristics

As of January 31, 2022, 182 cases and 441 controls consented to take part in the CLOCS. As shown in the CONSORT diagram in Figure 1, one case was excluded for providing insufficient consent and 14 control participants withdrew or were excluded. Of the remaining 427 controls, only 160 (37.5%) provided sufficient ID verification and were therefore eligible for loyalty card data requests. There were 4 cases who completed the risk factor questionnaire but did not provide loyalty card details and therefore were excluded from purchase history requests.

The characteristics of CLOCS participants are shown in Table 1. On average, cases were about 13 years older than controls. In terms of loyalty card ownership, 47% of cases and 33% of controls owned cards from both retailers, while the remaining participants had one or the other. The majority of cases who took part in the CLOCS were diagnosed with serous ovarian cancer (75.8%) and of those, 94% were diagnosed at stage 3 or 4 (see Table S1 in Multimedia Appendix 1 for details).

Ovarian cancer risk and risk scores among participants are shown in Table S2 and Table S3, respectively, of Multimedia Appendix 1. The majority of cases (46.7%) fell into the high ovarian cancer risk score tertile, while 24.7% had a medium risk score and 28.6% of cases were in the low risk score tertile. 

Purchase histories were requested for 178 cases and 160 controls from HSR1 and HSR2, as described in Figure 1. After consent, ID verification, matching loyalty card details with retailers’ records, and recontacting participants who did not match to clarify loyalty card details, purchase histories from 173 (97.2%) cases and 138 (86.3%) controls were transferred to the CLOCS team for analysis. There were 5 (2.8%) cases and 22 (13.8%) controls whose loyalty card details did not match the records at either HSR1, HSR2, or both. There were 20 cases and 18 controls with a previous cancer diagnosis before participating in the study and were excluded from all purchase history analyses (participants with a previous nonmelanoma skin cancer were included). After exclusion of participants with a previous cancer diagnosis and matching cases and controls on age and cardholder status, purchase histories of 153 cases and 306 controls (77 unique individuals) were included in purchase history analyses. The median age difference for matched pairs was 0.3 years (IQR –0.4 to 1.7 years). Among this reduced set of participants, only OC use was associated with ovarian cancer risk and was therefore the only risk factor adjusted for in subsequent purchase history analyses.

**Table 1:** Characteristics of participants.

Characteristic		Cases (n=182)	Controls (n=427)
Age (years), mean (SD)^a^		64.7 (10.9)	51.6 (13.7)
**Age group (years), n (%)**			
	18-39	6 (3.6)	61 (16.2)
	40-49	7 (4.7)	96 (23.8)
	50-59	32 (21.3)	125 (31.8)
	60-69	64 (36.7)	107 (21.2)
	≥70	73 (33.7)	33 (5.9)
**Ethnicity, n (%)**			
	White	173 (95.1)	400 (93.7)
	Nonwhite	8 (4.4)	11 (2.6)
	Prefer not to say	0 (0)	0 (0)
	Missing	1 (0.5)	16 (3.7)
**Loyalty card, n (%)**			
	HSR1^b^ card only	48 (26.4)	169 (39.6)
	HSR2^c^ card only	44 (24.2)	116 (27.2)
	Both HSR1 and HSR2	86 (47.3)	142 (33.3)
	Neither	4 (2.2)	0 (0)
**Loyalty card use, n (%)**			
	Not at all	1 (0.5)	2 (0.5)
	Not very often	9 (4.9)	10 (2.3)
	Sometimes	18 (9.9)	42 (9.8)
	Often	42 (23.1)	117 (27.4)
	All the time	109 (59.9)	256 (60.0)
	Missing	3 (1.6)	0 (0)
**Number of members in the household (including participant)^d^, n (%)**			
	1	32 (22.2)	43 (14.5)
	2	75 (52.1)	120 (40.5)
	≥3	29 (20.1)	131 (44.3)
	Missing	8 (5.6)	2 (0.7)

^a^Missing data for n=5 (0.9%) controls.

^b^HSR1: high street retailer 1.

^c^HSR2: high street retailer 2.

^d^Number of members in the household question was added to the questionnaire after amendment and during active recruitment. Missingness describes those who participated before the question was available.

### Cumulative Purchases

**Figure 2:**
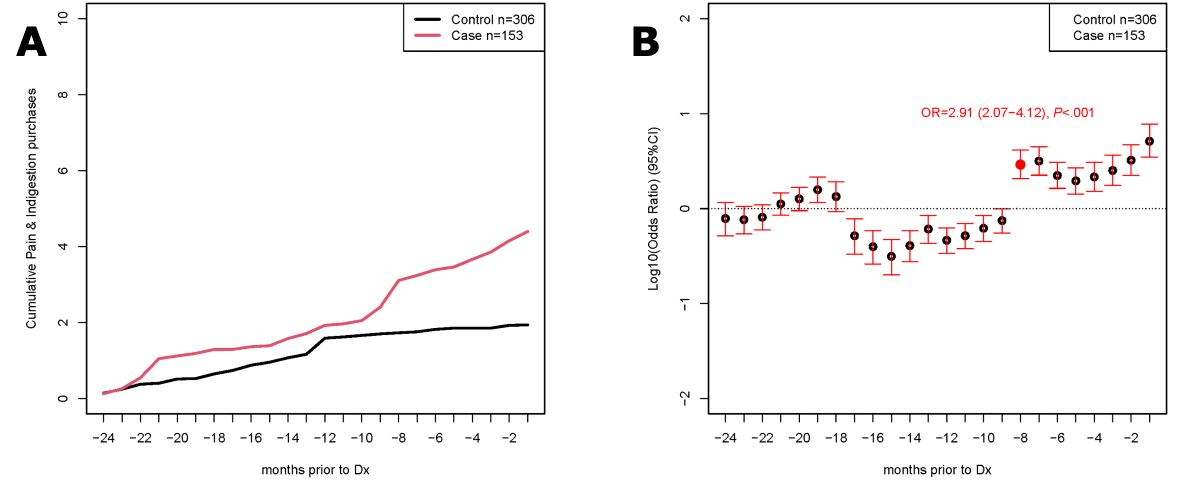
Combined pain and indigestion medications in the Cancer Loyalty Card Study (CLOCS). Cases (n=153) were matched with controls with respect to age and store card use (HSR1 only, HSR2 only, or HSR1+HSR2) at a 2:1 ratio with replacement (n=306 controls in matched analysis, 77 unique individuals). Participants with a previous diagnosis of cancer were excluded. Where possible, the same 24 months of purchases from controls were aligned to each matched case leading to their diagnosis (–24 months to –1 month on the X-axis); otherwise, the last 24 months of purchases were used for controls. (A) Average cumulative purchases of combined pain and indigestion medications in cases (red) and controls (black). (B) Purchases summed on a sliding 3-month window for cases and controls comparing all purchases to purchase of both pain and indigestion medication bought together. The Fisher exact test was used to calculate the odds ratios and significance of enrichment at each month prior to diagnosis. Dx: diagnosis; HSR: high street retailer.

The average cumulative number of purchases of pain and indigestion medications together are shown in Figure 2A. Throughout the 24 months, there was an increased number of these purchases among cases before diagnosis compared with that of controls, with an increased rate of purchases 8 months before diagnosis. The mean numbers of purchases of pain and indigestion medications were greater for cases in the 6 months, 12 months, and 24 months before diagnosis, with a significant association between these purchases and ovarian cancer diagnosis in the 6 months before diagnosis (OR 1.05, 95% CI 1.01-1.10; Table S4 in Multimedia Appendix 1). The Fisher exact test (Figure 2B) showed maximum discrimination between cases and controls 8 months before diagnosis (OR 2.91, 95% CI 2.07-4.12).

### Adjusted Models

**Figure 3:**
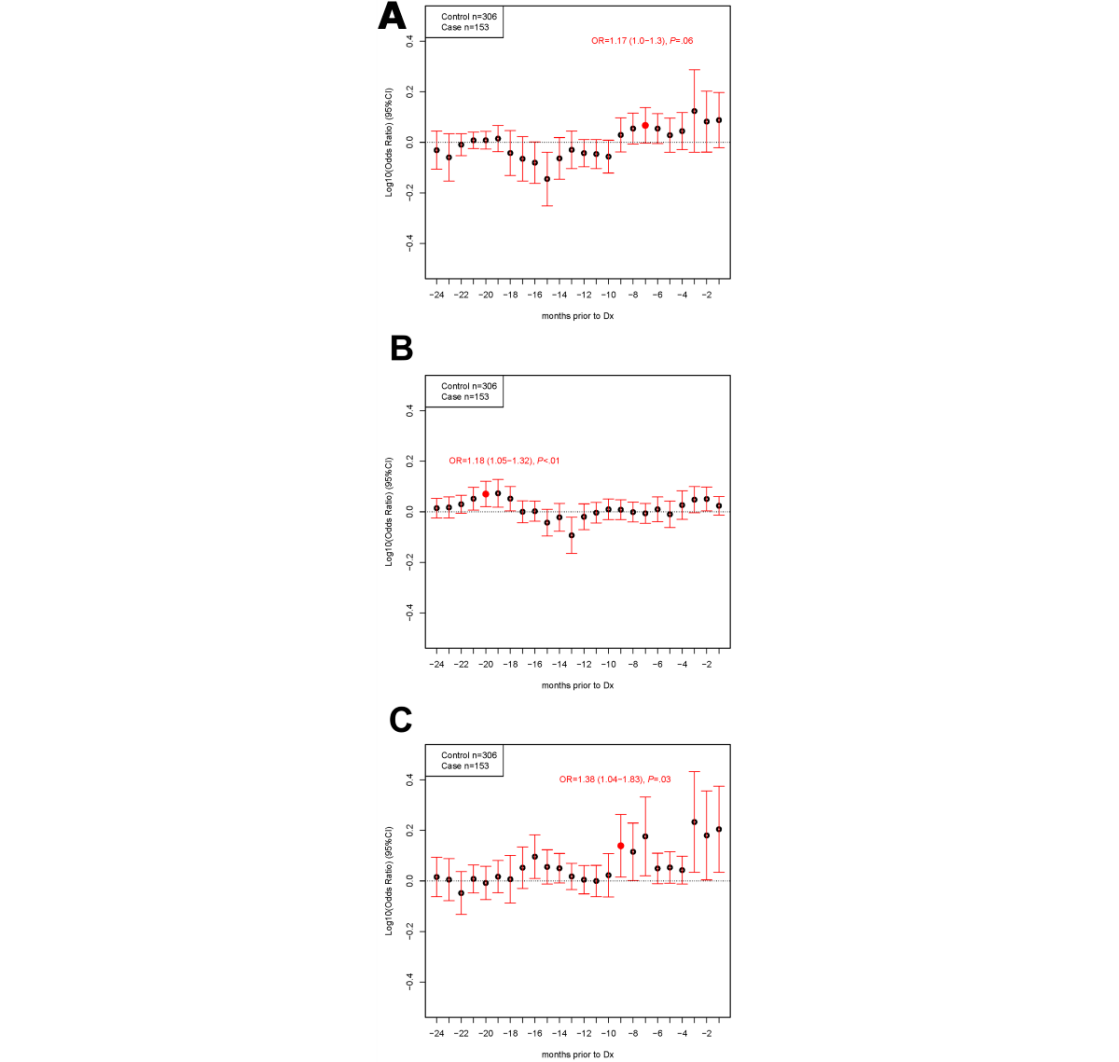
Conditional logistic regression for purchases prior to diagnosis (Dx). Cases (n=153) were matched with controls according to age and store card use (HSR1 only, HSR2 only, or HSR1+HSR2) at a 2:1 ratio with replacement (n=306 controls in matched analysis, 77 unique individuals). Participants with a previous diagnosis of cancer were excluded. Where possible, the same 24 months of purchases from controls were aligned to each matched case leading to their diagnosis (–24 months to –1 month on the X-axis); otherwise, the last 24 months of purchases were used for controls. Conditional logistic regression of matched case-control sets adjusting for oral contraceptive pill use and household number for combined sum of pain and indigestion medication (A), pain medication only (B), or indigestion medication only (C). HSR: high street retailer; OR: odds ratio.

As shown in Figure 3A, the likelihood of being diagnosed with ovarian cancer was significantly lower at 15 months before diagnosis in combined purchases of pain and indigestion medications, and there was no significant increase in risk of ovarian cancer diagnosis. However, when pain medication purchases were considered alone, there was a significantly increased likelihood of ovarian cancer 19 months before diagnosis (OR 1.18, 95% CI 1.05-1.32), as shown in Figure 3B. There was a significantly increased association between ovarian cancer diagnosis and indigestion medication purchases alone 9 months before diagnosis (OR 1.38, 95% CI 1.04-1.83), as shown in Figure 3C and Table S5 of Multimedia Appendix 1.

### ROC Analyses

**Figure 4:**
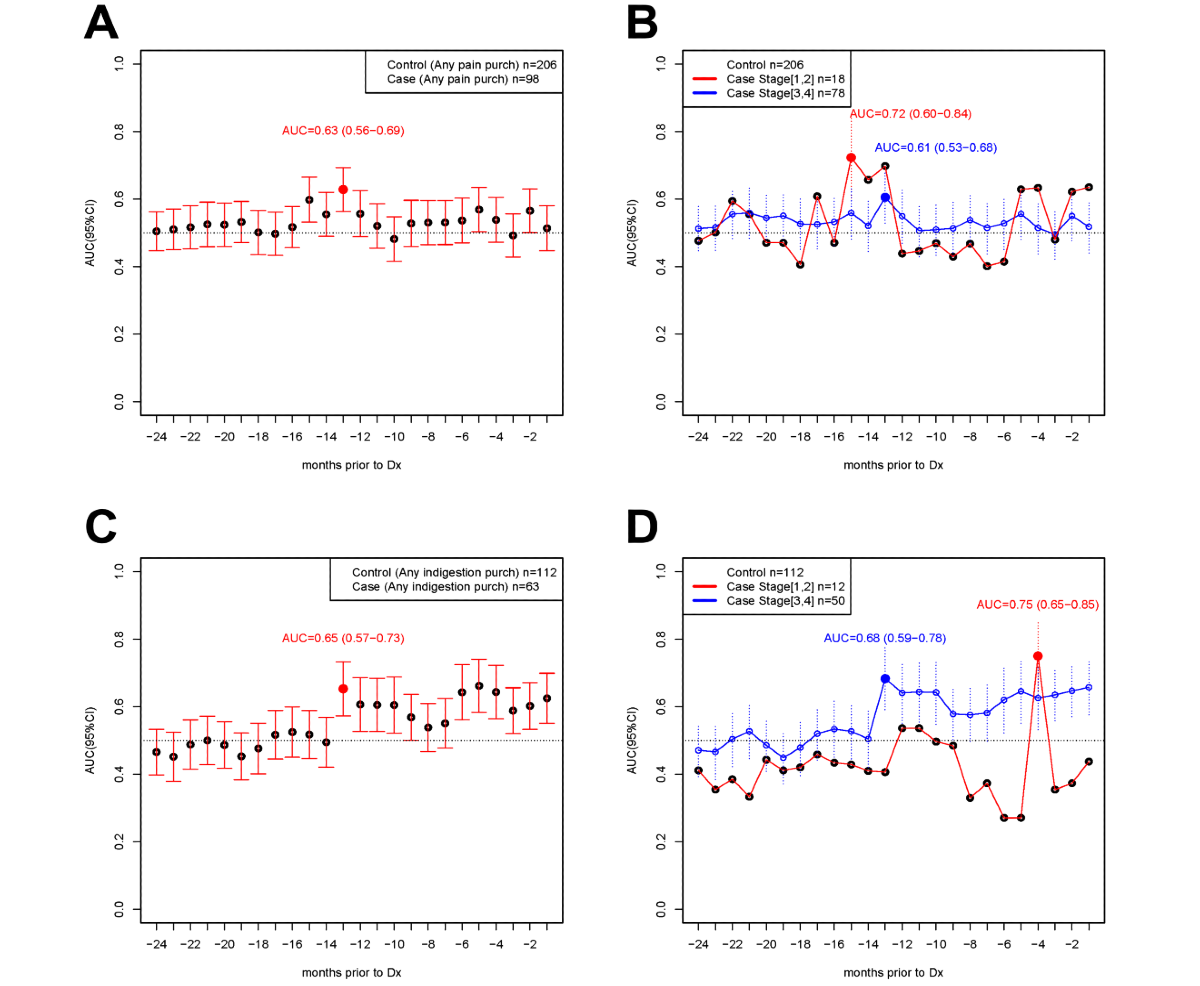
Receiver operating characteristic (ROC) analysis stratifying by stage of diagnosis (Dx). Purchases in cases and controls were aligned as per Figure 3. (A) ROC analysis of pain medications at each month prior to diagnosis in cases (n=98) and controls (n=206) that purchased any pain medication in the 24-month period. Area under the curve (AUC) and 95% CIs are presented. (B) Stratification into cases diagnosed at a low stage (1,2) (n=18, red line) or high stage (3,4) (n=78, blue line). (C) ROC analysis of indigestion medications at each month prior to diagnosis in cases (n=63) and controls (n=112) that purchased any indigestion medication in the 24-month period. AUC and 95% CIs are presented. (D) Stratification into cases diagnosed at a low stage (1,2) (n=12, red line) or high stage (3,4) (n=50, blue line).

Restricting the analysis to only participants who purchased any pain medication over the 24-month period, 98 cases and 206 controls remained. The ROC analysis for pain medication purchases alone (Figure 4A and Table S6 in Multimedia Appendix 1), excluding participants who did not purchase any pain medication, showed a maximum area under the curve (AUC) at 13 months before diagnosis (AUC=0.63, 95% CI 0.56-0.69). When further stratified on early stage at diagnosis, the AUC reached a maximum of 0.72 (95% CI 0.60-0.84) 15 months before diagnosis (Figure 4B).

Among those who only purchased any indigestion medication over the 24-month period, 63 cases and 112 controls remained. The ROC analysis for indigestion medication purchases alone (Figure 4C and Table S6 of Multimedia Appendix 1) also showed a maximum AUC at 13 months before diagnosis (AUC=0.65, 95% CI 0.57-0.73), and this further improved when restricted to late-stage ovarian cancer diagnosis (AUC=0.68, 95% CI 0.59-0.78), as shown in Figure 4D. Overall, the AUC was greater for late-stage diagnosis than early-stage diagnosis in all 24 months before diagnosis.

### Sensitivity Analyses

Adjusting for purchase behaviors before and after the COIVD-19 pandemic, different seasons, number of household members, and OC use showed little to no effect on the association of purchases with ovarian cancer over 24 months before diagnosis (Tables S7-S9 in Multimedia Appendix 1).

After adjustment for the ovarian cancer risk score (Tables S10-S12 in Multimedia Appendix 1), there was no significant change in the OR for detecting cancer symptoms among CLOCS participants who purchased pain and indigestion medications compared with only adjusting for OC use and number of household members.

## Discussion

### Principal Results

The primary aim of the CLOCS was to investigate purchasing behaviors of women with and without ovarian cancer before diagnosis to determine whether there is a difference in purchases of OTC medications among ovarian cancer patients before diagnosis. Purchases among CLOCS participants indicated an increase of pain and indigestion medication purchases prior to diagnosis of ovarian cancer. Specifically, we detected an increase in purchases of pain and indigestion medications bought together at 8 months before diagnosis. When considered separately in conditional logistic regression models adjusting for confounders, the signal for increased purchases of indigestion medications occurred from 9 months before diagnosis. For pain medication, we observed a period of increased purchases at 19 months prior to diagnosis, but no significant differences in the 12 months leading up to diagnosis. This suggests that there may be a difference in the timeline along which the patients with ovarian cancer included in this study may have experienced and cared for symptoms that can be treated with pain or indigestion medication.

### Comparison With Prior Work and Clinical Implications

The guidelines for investigation and initial assessment of ovarian cancer in symptomatic women vary greatly internationally partially due to the nonspecific nature of symptoms [12], and almost 1 in 4 cancer patients present with a broad range of abdominal symptoms before diagnosis [13]. Specifically, for an effective treatment of ovarian cancer, while symptom presentation may not always correlate with early-stage diagnosis, an earlier diagnosis even at stage IV could mean that patients may receive treatments that could improve their chances of surviving 1 year or longer. In the context of this study, when stratified by stage of diagnosis, for those diagnosed at an early stage, we identified that the purchase of pain medications occurred at 15 months before diagnosis, whereas indigestion medications were much more closely associated to diagnosis, at 4 months. For those diagnosed at a late stage, we identified indigestion medication purchases as early as 13 months prior to diagnosis and then in every month from 6 months up to diagnosis, but no significant change in pain medication purchases was found in the 12 months prior to diagnosis. From a clinical point of view, this highlights a unique interval in the tumor development where symptoms are presenting at different times. However, our sample sizes were too small to further stratify according to patient characteristics based on their OTC use associated with reported symptoms and stage at diagnosis.

While these differences could reflect the nonspecific nature of symptoms that occur during the development of ovarian cancer, they could also indicate the complex symptom appraisal processes for nonspecific cancer symptoms and presentation in health care [14]. For example, patients with pain 1 year before diagnosis might have already seen a health care professional following the initial presentation of their symptoms. However, in the absence of other symptoms not warranting an urgent cancer referral (ie, diagnostic pathway in the United Kingdom where patients with suspected cancer are seen within 2 weeks of presentation), patients’ symptoms may have been treated for other illnesses, resulting in a longer diagnostic interval from the first onset of symptoms [15]. In fact, we identified no differences in the number of GP visits in the 12 months leading up to diagnosis or participation in the study between cases and controls, indicating that transactional data could be useful to further explore self-care behaviors even after patients may have presented in health care for their symptoms. A previous study reported that 36% of ovarian cancer patients presented to their GP at least three times before diagnosis [16], while approximately 25.8% of cases in the CLOCS reported attending three or more visits to the GP before diagnosis (see Table S3 in Multimedia Appendix 1). Much lower rates of help-seeking behaviors could be attributed to the fact that approximately 64% of cases were diagnosed with ovarian cancer throughout the COVID-19 pandemic, during which many people avoided contacting or seeking help about their symptoms from their local GP [17]. Our results further emphasize the importance of safety nets to be in place between community pharmacies and primary care to ensure that patients who continue to care for their ongoing symptoms are identified and referred promptly.

Alternatively, the duration and the effectiveness of self-care using OTC indigestion and pain medication may be related to the type of abdominal symptom experienced [13]. However, there is little qualitative evidence on patients’ accounts of when they experienced pain and indigestion, or how they coped with these symptoms and cared for them before they received a diagnosis [18]. Nevertheless, the number of cases in the CLOCS with early-stage diagnosis was small; therefore, further studies with a larger number of patients with early-stage diagnosis would be needed to verify if self-care behaviors and earlier presentation are moderated by symptom development and individuals’ symptom appraisal processes.

### Strengths and Limitations

The main strength of this study is that this is the first study to investigate transactional data for understanding individuals’ self-care behaviors prior to a cancer diagnosis using a case-control study design. The use of prospectively collected data prior to cancer diagnosis avoids potential recall bias that would have occurred from querying as to when patients purchased OTC products for nonspecific symptoms using questionnaires. In the CLOCS, exposure to pain and indigestion medication purchases was measured using loyalty card data that are recorded by retailers, thereby adjusting for the potential recall bias as well as respondents’ bias to provide desirable outcomes. For example, although there was a significant difference between purchases of indigestion medications among cases and controls, the self-reported indigestion symptoms were not significantly different between cases and controls in this study (see Table S13 in Multimedia Appendix 1). Recall bias is a common issue reported for case-control studies; thus, to overcome recall bias associated with symptoms experienced before diagnosis, we aimed to recruit patients soon after their cancer diagnosis and/or exclude patients diagnosed before 2018.

One of the main limitations of this study is that purchase of an item is not the same as consumption of the item. The majority of participants reported that they lived with others in their household, and therefore could have been purchasing pain and indigestion medications for others instead of directly consuming these themselves. This limitation is applied equally to cases and controls who might also be buying products for others. There was no difference in the effect of purchases and ovarian cancer diagnosis when adjusting for other household members. Similarly, the analysis was restricted to purchases recorded only when participants used their loyalty cards, and any items purchased without the loyalty card were not recorded. However, 85% of participants reported that they used their loyalty card often or all the time. It is also likely that participants could be shopping for these medications at other retailers not included in this study, resulting in an underrepresentation of purchases with the limited participating retailers. Therefore, it is possible that the significant association observed here could be stronger with the inclusion of purchases of these items from other retailers, although further studies including more retailers are needed to verify this possibility.

Furthermore, most of the timeline for recruitment of cases and controls to this case-control study occurred during the COVID-19 pandemic. As a result of the paused recruitment and necessary amendments, the study did not meet the original goal to recruit 500 cases and 500 controls, and the sample size for analysis was smaller than originally intended. Due to the nature of recruitment of cases and controls, there was on average a 13-year age difference between cases and controls, reflecting an age bias in acceptability of sharing data, participating in research, and online recruitment of controls (HRB, YH, and JMF preliminary data, August 8, 2022). However, this age difference was accounted for in all analyses either through age adjustment or matching on age.

### Future Research

This study highlights the significant timeframe (9 to 19 months prior to diagnosis) in the patient interval in cancer diagnosis for when individuals who have yet to receive an ovarian cancer diagnosis and may have been reacting to the bodily changes and caring for their symptoms using OTC medications to maintain their health status. In the landscape of cancer epidemiology and symptom presentation research [5,6], this result contradicts with the timelines reported for the first symptoms recorded on primary care–based records to calculate the patient interval, which potentially highlights the need to have better data collection methods on patient well-being.

Likewise, this study only reported the temporal associations between OTC purchases and ovarian cancer diagnosis, and future data analyses will include understanding the characteristics of changing patterns of purchases, as well as combining other purchase categories that are not linked to symptom management using unbiased machine-learning approaches. Future analysis may also consider the association between OTC purchases and other diseases with similar symptoms, such as gastrointestinal diseases. With growing literature on utilizing other data sources in health research, transactional data have already been used to evaluate dietary patterns, and such analysis is considered to be a tool that can supplement our understanding of people’s dietary behaviors [19,20]. Similarly, this type of surveillance could provide an opportunity to utilize loyalty card programs, where available around the world, to assess purchases related to OTC treatment of many cancer types to improve earlier detection.

### Conclusions

To date, the CLOCS has identified an increase in purchases of OTC pain and indigestion medications among ovarian cancer patients before diagnosis compared with those of women without ovarian cancer. Further studies with larger numbers of ovarian cancer patients, diagnosed at different stages, and more participating retailers are needed to verify these findings, which can lead to the future development of an alert system for individuals to seek medical attention for the symptoms they are experiencing sooner than they might otherwise.

## Appendix

Multimedia Appendix 1 Supplementary tables.

## Abbreviations

AUC: area under the curve
CLOCS: Cancer Loyalty Card Study
CONSORT: Consolidated Standards of Reporting Trials
GP: general practitioner
HRT: hormone replacement therapy
HSR1: high street retailer 1
HSR2: high street retailer 2
NHS: National Health Service
OC: oral contraceptive
OR: odds ratio
OTC: over the counter
ROC: receiver operating characteristic

## Data Availability

Access to all Cancer Loyalty Card Study (CLOCS) purchase history data collected from the retailers is restricted due to the data sharing agreements in place and the sensitive nature of the data. However, we welcome other researchers to contact us for collaborations. The principal investigator and research team will review each proposal and, if approved, the analysis will be conducted by a member of the CLOCS team.
