# Supplementary material for: Association Between Purchase of Over-the-Counter Medications and Ovarian Cancer Diagnosis in the Cancer Loyalty Card Study (CLOCS): Observational Case-Control Study
Source: JMIR Public Health Surveill. 2023 Jan 26;9:e41762. doi: 10.2196/41762 (PMC9912145; doi:10.2196/41762)
Supplement: Multimedia Appendix 1 [file publichealth_v9i1e41762_app1.docx]

**Multimedia Appendix 1 Supplementary tables.**

**Supplementary Results**

Table S1: Diagnosis stage and histological subtype characteristics for cases in CLOCS.

| **Stage at Diagnosis** | **Serous*** | | **Nonserous**** | | **Missing** | | **Total** | |
| --- | --- | --- | --- | --- | --- | --- | --- | --- |
|  | **N** | **%** | **N** | **%** | **N** | **%** | **N** | **%** |
| Early (1-2) | 12 | 26.7 | 32 | 71.1 | 1 | 2.2 | 45 | 100.0 |
| Late (3-4) | 126 | 94.0 | 8 | 6.0 | 0 | 0.0 | 134 | 100.0 |
| Missing | 0 | 0.0 | 1 | 33.3 | 2 | 66.7 | 3 | 100.0 |
| Total | 138 | 75.8 | 41 | 22.5 | 3 | 1.6 | 182 | 100.0 |

Among all CLOCS participants (cases N=182, controls N=427), age (OR: 1.10, 95% CI: 1.08, 1.12), menopausal status (OR: 9.35, 95% CI: 4.43, 19.72), and HRT use (OR: 1.93, 95% CI: 1.30, 2.85) were significantly associated with an increased risk of ovarian cancer, shown in supplementary table S2. OC use was significantly associated with a decreased risk of ovarian cancer (OR: 0.43, 95%CI: 0.27, 0.67). After adjustment of these factors where appropriate, age and menopausal status remained significantly associated with an increased risk of ovarian cancer (OR: 1.10, 95% CI: 1.05, 1.10; OR: 2.52, 95% CI: 1.06, 5.98, respectively), and OC use remained significantly associated with a decreased risk of ovarian cancer (OR: 0.44, 95%CI: 0.25, 0.79). *Serous includes High Grade Serous Ovarian Cancer (HGSOC) (n=131) and Low Grade Serous Ovarian Cancer (n= 7). ** Non-Serous included Endometrioid (n=13), Clear Cell (N=14), Mucinous (n=5), borderline (n=5) and Other (n=4).

Table S2: Odds ratio of ovarian cancer diagnosis among CLOCS participants using unconditional logistic regression.

| **Risk factor** | **Cases (n=182)** | **Controls (n=427)** | **Unadjusted OR^1^** | **95% CI^1^** | ***P*** | **Adjusted OR^1,2^** | **95% CI^1,2^** | ***P**** |
| --- | --- | --- | --- | --- | --- | --- | --- | --- |
| **Age (years)** | 182 | 422 | 1.10 | 1.08, 1.12 | <0.001 | 1.10 | 1.05, 1.10 | <0.001 |
| Missing | 0 | 5 |  |  |  |  |  |  |
|  |  |  |  |  |  |  |  |  |
| **OC use** |  |  |  |  |  |  |  |  |
| Never | 43 | 50 | 1.00 | baseline |  | 1.00 | baseline |  |
| Ever | 138 | 377 | 0.43 | 0.27, 0.67 | <0.001 | 0.44 | 0.25, 0.79 | 0.005 |
| Missing | 1 | 0 |  |  |  |  |  |  |
|  |  |  |  |  |  |  |  |  |
| **Menopausal status** |  |  |  |  |  |  |  |  |
| Premenopausal | 8 | 106 | 1.00 | baseline |  | 1.00 | baseline |  |
| Postmenopausal | 163 | 231 | 9.35 | 4.43, 19.72 | <0.001 | 2.52 | 1.06, 5.98 | 0.036 |
| Missing | 11 | 90 |  |  |  |  |  |  |
|  |  |  |  |  |  |  |  |  |
| **HRT use** |  |  |  |  |  |  |  |  |
| Never | 123 | 343 | 1.00 | baseline |  | 1.00 | baseline |  |
| Ever | 58 | 84 | 1.93 | 1.30, 2.85 | 0.001 | 0.98 | 0.62, 1.55 | 0.933 |
| Missing | 1 | 0 |  |  |  |  |  |  |

^1^OR = Odds Ratio, CI = Confidence Interval

^2^Adjusted for age, oral contraceptive use, menopausal status, and hormone replacement therapy use, where appropriate

Table S3: Risk score results

| **Risk Score Tertile** | **Cases** | | **Controls** | |
| --- | --- | --- | --- | --- |
|  | **N** | **%** | **N** | **%** |
| Low | 52 | 28.6 | 142 | 33.3 |
| Medium | 45 | 24.7 | 142 | 33.3 |
| High | 85 | 46.7 | 143 | 33.5 |
| Total | 182 | 100.0 | 427 | 100.0 |

|  | **Cases** | | **Controls** | | **OR*** | **95% CI** |
| --- | --- | --- | --- | --- | --- | --- |
|  | N | Mean (SD) purchase per participant | N | Mean (SD) purchase per participant |  |  |
| Pain + Indigestion purchases over 6 month period | 153 | 3.2 (8.4) | 306 | 2.0 (3.0) | **1.05** | (1.01,1.10) |
|  |  |  |  |  |  |  |
| Pain purchases over 6 month period | 153 | 2.2 (5.2) | 306 | 1.6 (2.7) | 1.04 | (0.98,1.10) |
|  |  |  |  |  |  |  |
| Indigestion purchases over 6 month period | 153 | 1.0 (5.4) | 306 | 0.3 (0.7) | 1.12 | (0.96,1.32) |
|  |  |  |  |  |  |  |
| Pain + Indigestion purchases over 12 month period | 153 | 5.9 (13.6) | 306 | 4.3 (6.3) | 1.02 | (1.00,1.04) |
|  |  |  |  |  |  |  |
| Pain purchases over 12 month period | 153 | 4.1 (8.0) | 306 | 3.5 (5.4) | 1.02 | (0.98,1.05) |
|  |  |  |  |  |  |  |
| Indigestion purchases over 12 month period | 153 | 1.9 (8.5) | 306 | 0.8 (1.4) | 1.06 | (0.98,1.15) |
|  |  |  |  |  |  |  |
| Pain + Indigestion purchases over 24 month period | 153 | 11.4 (25.0) | 306 | 8.5 (11.4) | 1.01 | (1.00,1.02) |
|  |  |  |  |  |  |  |
| Pain purchases over 24 month period | 153 | 7.9 (15.7) | 306 | 6.4 (9.0) | 1.01 | (0.99,1.03) |
|  |  |  |  |  |  |  |
| Indigestion purchases over 24 month period | 153 | 3.5 (15.1) | 306 | 2.0 (3.9) | 1.02 | (1.00,1.04) |

Table S4: In support of Figure 2a and 2b. *OR and 95% confidence intervals from conditional logistic regression of cumulative purchase counts over 6, 12 months or 24 months prior to diagnosis adjusting for household number and oral contraceptive pill use.

Table S5: Conditional logistic regression analysis for purchase of pain and indigestion medication each month over 24 months before diagnosis and ovarian cancer risk.

|  | **Pain** | | **Indigestion** | |
| --- | --- | --- | --- | --- |
| **Months before Dx** | **OR** | **95% CI** | **OR** | **95% CI** |
| **1** | 1.07 | 0.99,1.16 | 1.60 | 1.08,2.37 |
| **2** | **1.12** | 1.02,1.24 | 1.51 | 1.01,2.27 |
| **3** | 1.09 | 0.98,1.21 | 1.71 | 1.08,2.71 |
| **4** | 1.03 | 0.92,1.16 | 1.10 | 0.97,1.25 |
| **5** | 0.97 | 0.88,1.09 | 1.13 | 0.98,1.31 |
| **6** | 1.01 | 0.91,1.12 | 1.12 | 0.98,1.29 |
| **7** | 0.98 | 0.89,1.07 | 1.50 | 1.05,2.15 |
| **8** | 0.98 | 0.90,1.08 | 1.30 | 1.00,1.69 |
| **9** | 1.01 | 0.93,1.11 | 1.37 | 1.04,1.83 |
| **10** | 1.03 | 0.94,1.12 | 1.05 | 0.87,1.28 |
| **11** | 1.00 | 0.91,1.09 | 0.99 | 0.87,1.15 |
| **12** | 0.98 | 0.88,1.09 | 1.01 | 0.89,1.15 |
| **13** | 0.86 | 0.74,1.00 | 1.04 | 0.92,1.17 |
| **14** | 0.99 | 0.88,1.11 | 1.12 | 0.98,1.28 |
| **15** | 0.96 | 0.86,1.07 | 1.13 | 0.97,1.33 |
| **16** | 1.04 | 0.96,1.14 | 1.25 | 1.02,1.52 |
| **17** | 1.04 | 0.95,1.14 | 1.13 | 0.93,1.36 |
| **18** | **1.12** | 1.02,1.25 | 1.02 | 0.82,1.26 |
| **19** | **1.15** | 1.03,1.28 | 1.04 | 0.90,1.20 |
| **20** | **1.15** | 1.03,1.28 | 0.98 | 0.84,1.14 |
| **21** | 1.09 | 1.00,1.20 | 1.01 | 0.90,1.16 |
| **22** | 1.06 | 0.98,1.15 | 0.90 | 0.74,1.09 |
| **23** | 1.03 | 0.94,1.13 | 1.01 | 0.84,1.23 |
| **24** | 1.03 | 0.95,1.13 | 1.04 | 0.87,1.24 |

Table S6: ROC analysis for purchase of pain medication or indigestion medications (Ind), respectively, each month over 24 months before diagnosis, and then stratified by stage at diagnosis. Early-stage is defined as diagnosis at stage 1 or 2, and late stage is defined as diagnosis at stage 3 or 4. (AUC 95% CI Lower Limit > 50 in bold, N = number of cases.)

| **Months before Dx** | **AUC (Pain)**  **(95% CI)**  **N=98** | **Early-stage AUC (Pain)**  **(95% CI)**  **N=18** | **Late-stage AUC (Pain)**  **(95% CI)**  **N=78** | **AUC (Ind.)**  **(95% CI)**  **N=63** | **Early-stage AUC (Ind.)**  **(95% CI)**  **N=12** | **Late-stage AUC (Ind.)**  **(95% CI)**  **N=50** |
| --- | --- | --- | --- | --- | --- | --- |
| **1** | 0.51 (0.45,0.58) | 0.64 (0.50,0.77) | 0.52 (0.44,0.60) | **0.62 (0.55,0.70)** | 0.44 (0.28,0.60) | **0.66 (0.58,0.74)** |
| **2** | 0.57 (0.50,0.63) | 0.62 (0.49,0.76) | 0.55 (0.48,0.62) | **0.60 (0.53,0.67)** | 0.37 (0.25,0.50) | **0.65 (0.57,0.72)** |
| **3** | 0.49 (0.43,0.56) | 0.48 (0.34,0.62) | 0.50 (0.42,0.57) | **0.59 (0.52,0.66)** | 0.35 (0.26,0.45) | **0.63 (0.56,0.71)** |
| **4** | 0.54 (0.47,0.60) | 0.63 (0.50,0.76) | 0.51 (0.44,0.59) | **0.64 (0.56,0.72)** | **0.75 (0.65,0.85)** | **0.63 (0.53,0.72)** |
| **5** | 0.57 (0.50,0.63) | 0.63 (0.50,0.76) | 0.56 (0.48,0.63) | **0.66 (0.58,0.74)** | 0.27 (0.17,0.37) | **0.65 (0.55,0.74)** |
| **6** | 0.54 (0.47,0.60) | 0.41 (0.28,0.55) | 0.53 (0.45,0.61) | **0.64 (0.56,0.72)** | 0.27 (0.17,0.37) | **0.62 (0.52,0.72)** |
| **7** | 0.53 (0.47,0.60) | 0.40 (0.27,0.53) | 0.51 (0.44,0.59) | 0.55 (0.48,0.62) | 0.37 (0.25,0.50) | 0.58 (0.50,0.66) |
| **8** | 0.53 (0.47,0.60) | 0.47 (0.33,0.61) | 0.54 (0.46,0.61) | 0.54 (0.47,0.61) | 0.33 (0.20,0.46) | 0.58 (0.50,0.65) |
| **9** | 0.53 (0.46,0.60) | 0.43 (0.29,0.57) | 0.51 (0.43,0.59) | 0.57 (0.50,0.64) | 0.48 (0.33,0.63) | 0.58 (0.50,0.65) |
| **10** | 0.48 (0.42,0.55) | 0.47 (0.33,0.61) | 0.51 (0.43,0.59) | **0.60 (0.52,0.69)** | 0.50 (0.31,0.68) | **0.64 (0.55,0.74)** |
| **11** | 0.52 (0.45,0.59) | 0.45 (0.31,0.58) | 0.51 (0.43,0.58) | **0.61 (0.53,0.68)** | 0.54 (0.36,0.71) | **0.64 (0.55,0.73)** |
| **12** | 0.56 (0.49,0.62) | 0.44 (0.30,0.58) | 0.55 (0.47,0.63) | **0.61 (0.53,0.69)** | 0.54 (0.35,0.72) | **0.64 (0.55,0.73)** |
| **13** | **0.63 (0.56,0.69)** | **0.70 (0.58,0.82)** | **0.61 (0.53,0.68)** | **0.65 (0.57,0.73)** | 0.41 (0.25,0.56) | **0.68 (0.59,0.78)** |
| **14** | 0.55 (0.49,0.62) | **0.66 (0.53,0.79)** | 0.52 (0.45,0.60) | 0.49 (0.42,0.57) | 0.41 (0.25,0.57) | 0.50 (0.42,0.59) |
| **15** | **0.60 (0.53,0.66)** | **0.72 (0.60,0.84)** | 0.56 (0.48,0.64) | 0.52 (0.45,0.59) | 0.43 (0.29,0.57) | 0.53 (0.45,0.61) |
| **16** | 0.52 (0.46,0.58) | 0.47 (0.34,0.60) | 0.53 (0.46,0.60) | 0.52 (0.45,0.60) | 0.43 (0.27,0.60) | 0.53 (0.45,0.62) |
| **17** | 0.50 (0.43,0.56) | 0.61 (0.48,0.74) | 0.53 (0.45,0.60) | 0.52 (0.44,0.59) | 0.46 (0.28,0.63) | 0.52 (0.44,0.60) |
| **18** | 0.50 (0.44,0.57) | 0.41 (0.27,0.54) | 0.53 (0.45,0.60) | 0.48 (0.40,0.55) | 0.42 (0.24,0.60) | 0.48 (0.40,0.56) |
| **19** | 0.53 (0.47,0.59) | 0.47 (0.33,0.61) | 0.55 (0.48,0.62) | 0.45 (0.38,0.52) | 0.41 (0.26,0.56) | 0.45 (0.37,0.53) |
| **20** | 0.52 (0.46,0.59) | 0.47 (0.32,0.62) | 0.54 (0.47,0.62) | 0.49 (0.42,0.56) | 0.44 (0.29,0.60) | 0.49 (0.41,0.56) |
| **21** | 0.53 (0.46,0.59) | 0.56 (0.40,0.71) | 0.56 (0.48,0.63) | 0.50 (0.43,0.57) | 0.33 (0.19,0.47) | 0.53 (0.45,0.61) |
| **22** | 0.52 (0.45,0.58) | 0.59 (0.45,0.74) | 0.56 (0.48,0.63) | 0.49 (0.42,0.56) | 0.39 (0.23,0.54) | 0.50 (0.42,0.59) |
| **23** | 0.51 (0.45,0.57) | 0.50 (0.37,0.64) | 0.52 (0.45,0.58) | 0.45 (0.38,0.52) | 0.35 (0.22,0.49) | 0.47 (0.38,0.55) |
| **24** | 0.50 (0.45,0.56) | 0.48 (0.35,0.61) | 0.51 (0.45,0.58) | 0.47 (0.40,0.53) | 0.41 (0.29,0.53) | 0.47 (0.39,0.55) |

Table S7: Sensitivity analysis results related to pain and indigestion medication purchase behaviours 1) before and after the COVID-19 pandemic (March 2020), 2) adjusting for seasonal purchases such as painkillers during cold and flu season, and 3) adjusting for the number of members in the household.

| **Months before Dx** | **Unadjusted**  **(n=153)** | **Pre-covid (N=58)** | **Post-covid**  **(N=95)** | **Seasonal**  **(N=153)** | **House. No. (N=153)** | **OC Use**  **N=153)** |
| --- | --- | --- | --- | --- | --- | --- |
|  | **OR (95% CI)** | **OR (95% CI)** | **OR (95% CI)** | **OR (95% CI)** | **OR (95% CI)** | **OR (95% CI)** |
| **1** | **1.10 (1.02,1.19)** | **1.13 (1.01,1.26)** | 1.06 (0.93,1.21) | **1.10 (1.01,1.18)** | **1.10 (1.02,1.19)** | **1.10 (1.01,1.19)** |
| **2** | **1.13 (1.03,1.24)** | 1.13 (1.00,1.28) | 1.14 (0.99,1.31) | **1.13 (1.03,1.25)** | **1.14 (1.04,1.25)** | **1.13 (1.03,1.25)** |
| **3** | **1.11 (1.01,1.22)** | 1.14 (0.98,1.31) | 1.08 (0.93,1.25) | **1.12 (1.01,1.25)** | **1.12 (1.01,1.24)** | **1.14 (1.02,1.27)** |
| **4** | 1.04 (0.97,1.12) | 1.08 (0.98,1.19) | 0.94 (0.80,1.10) | 1.05 (0.98,1.13) | 1.05 (0.98,1.13) | 1.07 (1.00,1.15) |
| **5** | 1.01 (0.94,1.09) | 1.05 (0.96,1.15) | 0.92 (0.80,1.07) | 1.01 (0.94,1.09) | 1.02 (0.95,1.1) | 1.04 (0.96,1.12) |
| **6** | 1.03 (0.96,1.10) | 1.05 (0.97,1.14) | 0.97 (0.84,1.12) | 1.03 (0.96,1.10) | 1.04 (0.97,1.11) | 1.05 (0.98,1.13) |
| **7** | 1.03 (0.97,1.10) | 1.04 (0.96,1.12) | 1.01 (0.90,1.14) | 1.03 (0.97,1.10) | 1.03 (0.97,1.10) | 1.04 (0.98,1.11) |
| **8** | 1.03 (0.96,1.10) | 1.03 (0.96,1.12) | 1.01 (0.88,1.15) | 1.02 (0.96,1.10) | 1.03 (0.96,1.10) | 1.04 (0.97,1.12) |
| **9** | 1.05 (0.98,1.14) | 1.07 (0.97,1.18) | 1.04 (0.92,1.17) | 1.05 (0.97,1.13) | 1.05 (0.98,1.14) | 1.06 (0.98,1.14) |
| **10** | 1.02 (0.95,1.10) | 1.13 (0.99,1.30) | 0.98 (0.89,1.07) | 1.03 (0.96,1.11) | 1.02 (0.95,1.10) | 1.02 (0.95,1.11) |
| **11** | 0.99 (0.93,1.06) | 1.13 (1.00,1.28) | 0.92 (0.83,1.02) | 0.96 (0.89,1.03) | 0.99 (0.93,1.06) | 1.00 (0.93,1.07) |
| **12** | 0.98 (0.92,1.05) | 1.06 (0.95,1.17) | 0.92 (0.82,1.02) | 0.97 (0.90,1.05) | 0.99 (0.92,1.06) | 0.98 (0.91,1.06) |
| **13** | 0.96 (0.88,1.05) | 1.02 (0.92,1.13) | 0.87 (0.76,1.00) | 0.96 (0.88,1.06) | 0.96 (0.88,1.05) | 0.94 (0.84,1.04) |
| **14** | 1.04 (0.96,1.12) | 1.03 (0.93,1.15) | 1.04 (0.93,1.17) | 1.03 (0.95,1.12) | 1.04 (0.96,1.12) | 1.02 (0.94,1.11) |
| **15** | 1.02 (0.94,1.10) | 1.04 (0.91,1.18) | 1.00 (0.90,1.11) | 1.01 (0.93,1.10) | 1.02 (0.93,1.10) | 0.98 (0.89,1.07) |
| **16** | 1.07 (0.99,1.15) | 1.07 (0.94,1.22) | 1.07 (0.98,1.17) | 1.06 (0.98,1.14) | 1.07 (1.00,1.16) | 1.04 (0.96,1.13) |
| **17** | 1.05 (0.98,1.14) | 1.03 (0.90,1.19) | 1.06 (0.97,1.16) | 1.03 (0.96,1.12) | 1.05 (0.98,1.14) | 1.02 (0.94,1.11) |
| **18** | **1.10 (1.01,1.19)** | 1.07 (0.93,1.23) | **1.11 (1.01,1.23)** | **1.11 (1.02,1.21)** | **1.10 (1.01,1.19)** | 1.08 (0.99,1.18) |
| **19** | **1.09 (1.01,1.18)** | 1.08 (0.97,1.19) | 1.11 (0.98,1.24) | **1.09 (1.01,1.17)** | **1.09 (1.01,1.18)** | **1.10 (1.01,1.20)** |
| **20** | 1.07 (1.00,1.15) | 1.06 (0.97,1.15) | 1.09 (0.99,1.21) | 1.06 (1.00,1.14) | 1.08 (1.00,1.15) | 1.08 (1.00,1.16) |
| **21** | 1.05 (0.99,1.12) | 1.05 (0.98,1.13) | 1.06 (0.95,1.18) | 1.05 (0.99,1.12) | 1.06 (1.00,1.13) | 1.07 (1.00,1.14) |
| **22** | 1.03 (0.97,1.10) | 1.03 (0.96,1.12) | 1.03 (0.93,1.15) | 1.04 (0.97,1.10) | 1.04 (0.98,1.10) | 1.03 (0.97,1.10) |
| **23** | 1.03 (0.96,1.11) | 1.05 (0.95,1.15) | 1.01 (0.91,1.13) | 1.02 (0.95,1.10) | 1.04 (0.97,1.11) | 1.03 (0.95,1.11) |
| **24** | 1.04 (0.97,1.11) | 1.03 (0.94,1.13) | 1.04 (0.94,1.14) | 1.04 (0.97,1.11) | 1.03 (0.97,1.11) | 1.03 (0.96,1.10) |

Table S8: Sensitivity analysis results related to pain medication purchase behaviours 1) before and after the COVID-19 pandemic (March 2020), 2) adjusting for seasonal purchases such as painkillers during cold and flu season, and 3) adjusting for the number of members in the household.

| **Months before Dx** | **Unadjusted** | **Pre-covid** | **Post-covid** | **Seasonal** | **Household Number** | **OC Use** |
| --- | --- | --- | --- | --- | --- | --- |
|  | **OR (95% CI)** | **OR (95% CI)** | **OR (95% CI)** | **OR (95% CI)** | **OR (95% CI)** | **OR (95% CI)** |
| **1** | 1.07 (0.99,1.16) | 1.11 (0.99,1.23) | 1.07 (0.99,1.16) | 1.07 (0.99,1.15) | 1.07 (0.99,1.16) | 1.06 (0.97,1.15) |
| **2** | **1.12 (1.02,1.24)** | 1.15 (1.00,1.31) | **1.12 (1.02,1.24)** | **1.12 (1.01,1.24)** | **1.12 (1.02,1.24)** | **1.12 (1.01,1.25)** |
| **3** | 1.09 (0.98,1.21) | 1.15 (0.99,1.33) | 1.09 (0.98,1.21) | 1.11 (0.99,1.24) | 1.10 (0.99,1.23) | 1.12 (0.99,1.25) |
| **4** | 1.03 (0.92,1.16) | 1.09 (0.93,1.27) | 1.03 (0.92,1.16) | 1.03 (0.92,1.16) | 1.05 (0.93,1.18) | 1.06 (0.93,1.21) |
| **5** | 0.98 (0.88,1.09) | 1.03 (0.90,1.17) | 0.98 (0.88,1.09) | 0.98 (0.87,1.09) | 0.99 (0.88,1.10) | 0.98 (0.87,1.10) |
| **6** | 1.01 (0.91,1.12) | 1.05 (0.92,1.20) | 1.01 (0.91,1.12) | 1.01 (0.91,1.13) | 1.02 (0.91,1.13) | 1.02 (0.91,1.14) |
| **7** | 0.98 (0.89,1.07) | 1.02 (0.92,1.12) | 0.98 (0.89,1.07) | 0.98 (0.89,1.07) | 0.98 (0.89,1.07) | 0.99 (0.90,1.08) |
| **8** | 0.98 (0.90,1.08) | 1.01 (0.92,1.12) | 0.98 (0.90,1.08) | 0.98 (0.89,1.07) | 0.98 (0.90,1.07) | 1.00 (0.91,1.09) |
| **9** | 1.02 (0.93,1.11) | 1.06 (0.95,1.19) | 1.02 (0.93,1.11) | 1.01 (0.93,1.11) | 1.02 (0.93,1.11) | 1.02 (0.93,1.12) |
| **10** | 1.03 (0.94,1.12) | 1.15 (0.98,1.36) | 1.03 (0.94,1.12) | 1.03 (0.94,1.13) | 1.03 (0.94,1.12) | 1.02 (0.93,1.12) |
| **11** | 1.00 (0.91,1.09) | 1.23 (1.01,1.48) | 1.00 (0.91,1.09) | 0.95 (0.86,1.05) | 1.00 (0.92,1.10) | 0.99 (0.90,1.09) |
| **12** | 0.98 (0.88,1.09) | 1.10 (0.92,1.31) | 0.98 (0.88,1.09) | 0.98 (0.87,1.10) | 0.99 (0.88,1.10) | 0.96 (0.85,1.07) |
| **13** | 0.86 (0.74,1.00) | 0.97 (0.77,1.22) | 0.86 (0.74,1.00) | 0.85 (0.73,0.99) | 0.86 (0.74,1.00) | **0.81 (0.68,0.95)** |
| **14** | 0.99 (0.88,1.11) | 0.97 (0.81,1.17) | 0.99 (0.88,1.11) | 0.98 (0.87,1.11) | 0.99 (0.88,1.11) | 0.95 (0.84,1.08) |
| **15** | 0.96 (0.86,1.07) | 0.87 (0.69,1.11) | 0.96 (0.86,1.07) | 0.96 (0.86,1.07) | 0.95 (0.85,1.07) | 0.91 (0.80,1.02) |
| **16** | 1.04 (0.96,1.14) | 1.01 (0.85,1.19) | 1.04 (0.96,1.14) | 1.03 (0.94,1.12) | 1.04 (0.96,1.14) | 1.01 (0.92,1.10) |
| **17** | 1.04 (0.95,1.14) | 0.99 (0.82,1.21) | 1.04 (0.95,1.14) | 1.01 (0.92,1.12) | 1.03 (0.94,1.14) | 1.00 (0.91,1.11) |
| **18** | **1.13 (1.02,1.25)** | 1.15 (0.98,1.35) | **1.13 (1.02,1.25)** | **1.15 (1.03,1.28)** | **1.13 (1.01,1.25)** | **1.13 (1.01,1.26)** |
| **19** | **1.15 (1.03,1.28)** | 1.16 (0.98,1.37) | **1.15 (1.03,1.28)** | **1.14 (1.02,1.27)** | **1.14 (1.02,1.28)** | **1.18 (1.04,1.34)** |
| **20** | **1.15 (1.03,1.28)** | 1.11 (0.97,1.27) | **1.15 (1.03,1.28)** | **1.13 (1.02,1.25)** | **1.15 (1.03,1.28)** | **1.18 (1.05,1.32)** |
| **21** | **1.09 (1.00,1.20)** | 1.06 (0.96,1.16) | **1.09 (1.00,1.20)** | **1.11 (1.00,1.22)** | 1.10 (1.00,1.21) | **1.13 (1.02,1.25)** |
| **22** | 1.06 (0.98,1.15) | 1.05 (0.96,1.15) | 1.06 (0.98,1.15) | 1.06 (0.98,1.14) | 1.06 (0.98,1.15) | 1.07 (0.99,1.16) |
| **23** | 1.03 (0.94,1.13) | 1.04 (0.93,1.18) | 1.03 (0.94,1.13) | 1.02 (0.93,1.12) | 1.04 (0.95,1.13) | 1.04 (0.95,1.14) |
| **24** | 1.03 (0.95,1.13) | 1.04 (0.92,1.18) | 1.03 (0.95,1.13) | 1.04 (0.95,1.13) | 1.03 (0.95,1.12) | 1.03 (0.95,1.13) |

Table S9: Sensitivity analysis results related to indigestion medication purchase behaviours 1) before and after the COVID-19 pandemic (March 2020), 2) adjusting for seasonal purchases such as painkillers during cold and flu season, and 3) adjusting for the number of members in the household.

| **Months before Dx** | **Unadjusted** | **Pre-covid** | **Post-covid** | **Seasonal** | **Household Number** | **OC Use** |
| --- | --- | --- | --- | --- | --- | --- |
|  | **OR (95% CI)** | **OR (95% CI)** | **OR (95% CI)** | **OR (95% CI)** | **OR (95% CI)** | **OR (95% CI)** |
| **1** | **1.76 (1.19,2.59)** | **1.80 (1.05,3.09)** | **1.71 (0.97,3.01)** | **1.79 (1.20,2.68)** | **1.76 (1.19,2.59)** | **1.60 (1.08,2.37)** |
| **2** | **1.61 (1.10,2.37)** | 1.51 (0.93,2.45) | 1.75 (0.98,3.13) | **1.64 (1.11,2.43)** | **1.66 (1.11,2.47)** | **1.51 (1.01,2.25)** |
| **3** | **1.74 (1.12,2.71)** | **1.93 (1.06,3.49)** | 1.50 (0.74,3.04) | **1.77 (1.13,2.78)** | **1.83 (1.16,2.89)** | **1.70 (1.08,2.68)** |
| **4** | 1.07 (0.95,1.20) | 1.18 (0.89,1.56) | 0.37 (0.17,0.79) | 1.09 (0.94,1.26) | 1.07 (0.95,1.21) | 1.10 (0.97,1.25) |
| **5** | 1.08 (0.94,1.24) | 1.14 (0.93,1.39) | 0.76 (0.47,1.26) | 1.07 (0.92,1.25) | 1.09 (0.94,1.25) | 1.13 (0.98,1.30) |
| **6** | 1.08 (0.94,1.24) | 1.16 (0.89,1.52) | 0.81 (0.52,1.25) | 1.06 (0.93,1.22) | 1.09 (0.94,1.25) | 1.12 (0.98,1.29) |
| **7** | **1.56 (1.08,2.26)** | 1.58 (0.92,2.71) | 1.55 (0.93,2.58) | **1.57 (1.07,2.31)** | **1.59 (1.09,2.33)** | **1.50 (1.05,2.14)** |
| **8** | **1.34 (1.01,1.78)** | 1.44 (0.92,2.27) | 1.24 (0.82,1.89) | **1.38 (1.02,1.86)** | **1.36 (1.01,1.82)** | 1.30 (1.00,1.69) |
| **9** | **1.40 (1.03,1.89)** | 1.36 (0.93,2.00) | 1.45 (0.91,2.29) | **1.42 (1.03,1.95)** | **1.42 (1.04,1.94)** | **1.37 (1.04,1.82)** |
| **10** | 1.03 (0.85,1.24) | 1.23 (0.93,1.62) | 0.80 (0.58,1.12) | 1.05 (0.87,1.28) | 1.04 (0.86,1.26) | 1.05 (0.87,1.28) |
| **11** | 0.96 (0.84,1.10) | 1.11 (0.94,1.31) | 0.73 (0.55,0.96) | 0.94 (0.81,1.08) | 0.97 (0.85,1.11) | 1.00 (0.87,1.15) |
| **12** | 0.98 (0.87,1.10) | 1.08 (0.94,1.25) | 0.75 (0.58,0.97) | 0.94 (0.83,1.08) | 0.98 (0.87,1.11) | 1.01 (0.89,1.15) |
| **13** | 1.02 (0.91,1.15) | 1.07 (0.93,1.23) | 0.82 (0.60,1.12) | 1.05 (0.93,1.18) | 1.03 (0.92,1.15) | 1.04 (0.92,1.17) |
| **14** | 1.14 (0.96,1.34) | 1.12 (0.93,1.35) | 1.17 (0.86,1.58) | 1.14 (0.95,1.36) | 1.14 (0.97,1.34) | 1.12 (0.98,1.28) |
| **15** | 1.16 (0.97,1.39) | 1.19 (0.95,1.50) | 1.10 (0.79,1.52) | 1.16 (0.96,1.40) | 1.17 (0.98,1.40) | 1.14 (0.97,1.33) |
| **16** | **1.29 (1.04,1.60)** | 1.32 (0.98,1.79) | 1.24 (0.88,1.73) | **1.29 (1.03,1.61)** | **1.31 (1.05,1.64)** | **1.24 (1.02,1.51)** |
| **17** | 1.18 (0.97,1.42) | 1.25 (0.94,1.64) | 1.09 (0.82,1.46) | 1.14 (0.94,1.38) | 1.19 (0.99,1.44) | 1.13 (0.93,1.36) |
| **18** | 1.10 (0.90,1.34) | 1.15 (0.85,1.57) | 1.06 (0.81,1.38) | 1.09 (0.89,1.33) | 1.11 (0.91,1.36) | 1.02 (0.82,1.26) |
| **19** | 1.04 (0.91,1.19) | 1.09 (0.92,1.29) | 0.82 (0.57,1.19) | 1.05 (0.92,1.21) | 1.05 (0.92,1.20) | 1.04 (0.90,1.20) |
| **20** | 0.99 (0.87,1.14) | 1.07 (0.91,1.26) | 0.83 (0.62,1.12) | 0.99 (0.86,1.14) | 1.00 (0.87,1.15) | 0.98 (0.84,1.14) |
| **21** | 1.02 (0.91,1.14) | 1.10 (0.94,1.28) | 0.83 (0.63,1.10) | 1.00 (0.89,1.13) | 1.03 (0.92,1.16) | 1.02 (0.90,1.16) |
| **22** | 0.97 (0.81,1.15) | 1.09 (0.87,1.37) | 0.83 (0.61,1.12) | 0.98 (0.82,1.18) | 0.98 (0.82,1.17) | 0.90 (0.74,1.09) |
| **23** | 1.07 (0.91,1.26) | 1.18 (0.94,1.49) | 0.8 (0.53,1.20) | 1.07 (0.90,1.27) | 1.09 (0.92,1.28) | 1.01 (0.84,1.22) |
| **24** | 1.08 (0.92,1.28) | 1.12 (0.92,1.37) | 0.93 (0.61,1.42) | 1.08 (0.91,1.28) | 1.09 (0.92,1.30) | 1.04 (0.87,1.24) |

Table S10: Sensitivity analysis results related to ovarian cancer symptom behaviours and pain and indigestion medication purchases 1) adjusting for the risk score, 2) adjusting for GP visits, and 3) adjusting for reported symptoms.

| **Months before Dx** | **Unadjusted** | **Adjusted for risk score** | **GP visit** | **No GP visit** | **No Symptoms reported** | **Symptoms reported** |
| --- | --- | --- | --- | --- | --- | --- |
|  | **OR (95% CI)** | **OR (95% CI)** | **OR (95% CI)** | **OR (95% CI)** | **OR (95% CI)** | **OR (95% CI)** |
| **1** | **1.10 (1.02,1.19)** | **1.09 (1.01,1.18)** | 1.08 (1.00,1.17) | 1.17 (0.91,1.50) | 1.16 (0.81,1.67) | **1.10 (1.01,1.19)** |
| **2** | **1.13 (1.03,1.24)** | **1.13 (1.03,1.25)** | 1.11 (1.00,1.22) | 1.13 (0.89,1.42) | 1.01 (0.69,1.50) | **1.14 (1.04,1.26)** |
| **3** | **1.11 (1.01,1.22)** | 1.11 (1.00,1.23) | 1.08 (0.98,1.18) | 1.07 (0.81,1.41) | 1.19 (0.80,1.76) | 1.11 (1.00,1.22) |
| **4** | 1.04 (0.97,1.12) | 1.03 (0.96,1.11) | 1.03 (0.96,1.11) | 0.82 (0.58,1.14) | 0.91 (0.62,1.35) | 1.05 (0.98,1.13) |
| **5** | 1.01 (0.94,1.09) | 1.00 (0.93,1.08) | 0.99 (0.91,1.08) | 0.96 (0.75,1.21) | 1.05 (0.83,1.32) | 1.01 (0.93,1.09) |
| **6** | 1.03 (0.96,1.10) | 1.03 (0.95,1.10) | 1.02 (0.95,1.09) | 1.01 (0.84,1.22) | 1.05 (0.81,1.36) | 1.03 (0.96,1.10) |
| **7** | 1.03 (0.97,1.10) | 1.03 (0.96,1.10) | 1.02 (0.95,1.10) | 1.03 (0.89,1.20) | 1.10 (0.87,1.39) | 1.03 (0.96,1.10) |
| **8** | 1.03 (0.96,1.10) | 1.03 (0.96,1.10) | 1.02 (0.94,1.10) | 1.00 (0.85,1.18) | 0.95 (0.61,1.49) | 1.03 (0.96,1.10) |
| **9** | 1.05 (0.98,1.14) | 1.06 (0.98,1.14) | 1.03 (0.95,1.12) | 1.04 (0.88,1.23) | 0.93 (0.61,1.41) | 1.06 (0.98,1.14) |
| **10** | 1.02 (0.95,1.10) | 1.02 (0.95,1.10) | 1.00 (0.91,1.09) | 1.01 (0.86,1.17) | 0.77 (0.50,1.18) | 1.05 (0.97,1.14) |
| **11** | 0.99 (0.93,1.06) | 0.99 (0.92,1.06) | 0.99 (0.92,1.07) | 0.94 (0.79,1.12) | 0.86 (0.65,1.13) | 1.01 (0.94,1.09) |
| **12** | 0.98 (0.92,1.05) | 0.98 (0.91,1.05) | 0.98 (0.91,1.07) | 0.91 (0.74,1.11) | 0.87 (0.67,1.13) | 1.00 (0.93,1.08) |
| **13** | 0.96 (0.88,1.05) | 0.96 (0.88,1.04) | 0.96 (0.87,1.06) | 0.82 (0.62,1.08) | 0.95 (0.74,1.22) | 0.96 (0.87,1.06) |
| **14** | 1.04 (0.96,1.12) | 1.03 (0.95,1.12) | 1.01 (0.92,1.10) | 1.03 (0.81,1.31) | 1.11 (0.88,1.38) | 1.03 (0.94,1.12) |
| **15** | 1.02 (0.94,1.10) | 1.02 (0.94,1.11) | 0.98 (0.88,1.10) | 1.00 (0.82,1.21) | 1.04 (0.83,1.31) | 1.01 (0.93,1.10) |
| **16** | 1.07 (0.99,1.15) | **1.09 (1.01,1.18)** | 1.06 (0.96,1.17) | 1.03 (0.91,1.17) | 1.06 (0.89,1.27) | 1.07 (0.99,1.16) |
| **17** | 1.05 (0.98,1.14) | 1.08 (0.99,1.17) | 1.05 (0.95,1.16) | 1.03 (0.91,1.16) | 0.99 (0.81,1.21) | 1.06 (0.98,1.16) |
| **18** | **1.10 (1.01,1.19)** | **1.11 (1.02,1.21)** | 1.08 (0.97,1.20) | 1.07 (0.94,1.22) | 1.03 (0.79,1.33) | **1.11 (1.01,1.20)** |
| **19** | **1.09 (1.01,1.18)** | **1.10 (1.01,1.19)** | 1.06 (0.97,1.16) | 1.11 (0.94,1.30) | 1.06 (0.74,1.50) | **1.09 (1.01,1.18)** |
| **20** | 1.07 (1.00,1.15) | 1.08 (1.00,1.16) | 1.05 (0.97,1.13) | 1.11 (0.95,1.29) | 1.19 (0.84,1.67) | 1.07 (1.00,1.14) |
| **21** | 1.05 (0.99,1.12) | 1.06 (0.99,1.13) | 1.04 (0.98,1.11) | 1.08 (0.91,1.27) | 1.10 (0.81,1.49) | 1.05 (0.99,1.12) |
| **22** | 1.03 (0.97,1.10) | 1.05 (0.98,1.12) | 1.04 (0.97,1.11) | 1.02 (0.89,1.18) | 1.11 (0.84,1.46) | 1.03 (0.97,1.10) |
| **23** | 1.03 (0.96,1.11) | 1.05 (0.97,1.13) | 1.03 (0.95,1.12) | 0.98 (0.82,1.16) | 1.04 (0.77,1.41) | 1.03 (0.96,1.11) |
| **24** | 1.04 (0.97,1.11) | 1.05 (0.97,1.12) | 1.04 (0.95,1.13) | 0.97 (0.83,1.14) | 1.07 (0.75,1.53) | 1.03 (0.96,1.11) |

Table S11: Sensitivity analysis results related to ovarian cancer symptom behaviours and pain medication purchases 1) adjusting for the risk score, 2) adjusting for GP visits, and 3) adjusting for reported symptoms.

| **Months before Dx** | **Unadjusted** | **Adjusted for risk score** | **GP visit** | **No GP visit** | **No Symptoms reported** | **Symptoms reported** |
| --- | --- | --- | --- | --- | --- | --- |
|  | **OR (95% CI)** | **OR (95% CI)** | **OR (95% CI)** | **OR (95% CI)** | **OR (95% CI)** | **OR (95% CI)** |
| **1** | 1.07 (0.99,1.16) | 1.07 (0.99,1.15) | 1.06 (0.98,1.15) | 1.12 (0.86,1.47) | 1.12 (0.71,1.76) | 1.07 (0.99,1.16) |
| **2** | **1.12 (1.02,1.24)** | **1.12 (1.01,1.24)** | 1.10 (0.98,1.22) | 1.12 (0.87,1.44) | 0.96 (0.59,1.57) | **1.13 (1.02,1.25)** |
| **3** | 1.09 (0.98,1.21) | 1.09 (0.98,1.21) | 1.05 (0.93,1.19) | 1.05 (0.78,1.4) | 1.06 (0.67,1.68) | 1.09 (0.98,1.22) |
| **4** | 1.03 (0.92,1.16) | 1.02 (0.91,1.15) | 0.98 (0.85,1.13) | 0.85 (0.57,1.27) | 0.83 (0.47,1.47) | 1.05 (0.93,1.18) |
| **5** | 0.98 (0.88,1.09) | 0.96 (0.86,1.07) | 0.89 (0.76,1.04) | 1.01 (0.78,1.30) | 0.86 (0.51,1.45) | 0.98 (0.88,1.1) |
| **6** | 1.01 (0.91,1.12) | 1.01 (0.90,1.13) | 0.96 (0.84,1.10) | 1.02 (0.83,1.25) | 0.95 (0.59,1.51) | 1.01 (0.91,1.13) |
| **7** | 0.98 (0.89,1.07) | 0.98 (0.89,1.07) | 0.95 (0.85,1.07) | 1.01 (0.86,1.19) | 1.01 (0.7,1.47) | 0.98 (0.89,1.07) |
| **8** | 0.98 (0.90,1.08) | 0.98 (0.90,1.08) | 0.96 (0.86,1.08) | 0.98 (0.81,1.17) | 0.91 (0.55,1.53) | 0.99 (0.90,1.08) |
| **9** | 1.02 (0.93,1.11) | 1.01 (0.93,1.11) | 0.97 (0.87,1.09) | 1.05 (0.88,1.25) | 0.79 (0.46,1.36) | 1.03 (0.94,1.13) |
| **10** | 1.03 (0.94,1.12) | 1.02 (0.93,1.12) | 0.97 (0.86,1.09) | 1.05 (0.88,1.26) | 0.72 (0.39,1.33) | 1.06 (0.96,1.17) |
| **11** | 1.00 (0.91,1.09) | 1.01 (0.92,1.10) | 0.98 (0.88,1.09) | 1.00 (0.83,1.21) | 0.75 (0.42,1.32) | 1.03 (0.93,1.13) |
| **12** | 0.98 (0.88,1.09) | 0.99 (0.89,1.11) | 0.94 (0.82,1.08) | 0.98 (0.77,1.24) | 0.80 (0.50,1.28) | 1.00 (0.89,1.13) |
| **13** | 0.86 (0.74,1.00) | 0.86 (0.74,1.00) | 0.77 (0.63,0.94) | 0.92 (0.66,1.27) | 0.86 (0.53,1.39) | 0.86 (0.73,1.01) |
| **14** | 0.99 (0.88,1.11) | 0.99 (0.88,1.11) | 0.89 (0.76,1.05) | 1.03 (0.78,1.36) | 1.19 (0.83,1.71) | 0.97 (0.85,1.09) |
| **15** | 0.96 (0.86,1.07) | 0.96 (0.85,1.07) | 0.83 (0.69,1.01) | 0.99 (0.80,1.21) | 1.05 (0.79,1.40) | 0.94 (0.84,1.06) |
| **16** | 1.04 (0.96,1.14) | 1.06 (0.97,1.16) | 1.01 (0.89,1.15) | 1.03 (0.89,1.19) | 1.09 (0.87,1.36) | 1.03 (0.94,1.13) |
| **17** | 1.04 (0.95,1.14) | 1.06 (0.96,1.16) | 0.98 (0.84,1.14) | 1.05 (0.91,1.21) | 1.03 (0.79,1.35) | 1.04 (0.94,1.14) |
| **18** | **1.13 (1.02,1.25)** | **1.15 (1.02,1.29)** | 1.09 (0.94,1.27) | 1.12 (0.95,1.31) | 1.22 (0.78,1.9) | **1.12 (1.01,1.24)** |
| **19** | **1.15 (1.03,1.28)** | **1.15 (1.02,1.29)** | 1.08 (0.95,1.24) | 1.19 (0.94,1.51) | 1.24 (0.78,1.96) | 1.14 (1.02,1.27) |
| **20** | **1.15 (1.03,1.28)** | **1.15 (1.03,1.30)** | 1.09 (0.97,1.22) | 1.21 (0.96,1.52) | 1.74 (0.87,3.47) | 1.13 (1.02,1.25) |
| **21** | 1.09 (1.00,1.20) | 1.09 (0.99,1.21) | 1.06 (0.97,1.16) | 1.18 (0.92,1.51) | 1.47 (0.89,2.42) | 1.08 (0.99,1.18) |
| **22** | 1.06 (0.98,1.15) | 1.07 (0.98,1.17) | 1.06 (0.97,1.16) | 1.06 (0.90,1.25) | 1.4 (0.91,2.17) | 1.05 (0.97,1.13) |
| **23** | 1.03 (0.94,1.13) | 1.05 (0.95,1.15) | 1.02 (0.91,1.14) | 1.01 (0.83,1.22) | 1.28 (0.79,2.07) | 1.03 (0.94,1.13) |
| **24** | 1.03 (0.95,1.13) | 1.04 (0.95,1.14) | 1.02 (0.90,1.15) | 0.99 (0.82,1.20) | 1.15 (0.74,1.79) | 1.03 (0.95,1.12) |

Table S12: Sensitivity analysis results related to ovarian cancer symptom behaviours indigestion medication purchases 1) adjusting for the risk score, 2) adjusting for GP visits, and 3) adjusting for reported symptoms.

| **Months before Dx** | **Unadjusted** | **Adjusted for risk score** | **GP visit** | **No GP visit** | **No Symptoms reported** | **Symptoms reported** |
| --- | --- | --- | --- | --- | --- | --- |
|  | **OR (95% CI)** | **OR (95% CI)** | **OR (95% CI)** | **OR (95% CI)** | **OR (95% CI)** | **OR (95% CI)** |
| **1** | **1.76 (1.19,2.59)** | **1.74 (1.16,2.59)** | 1.54 (0.97,2.43) | 1.87 (0.80,4.34) | 1.91 (0.62,5.92) | **1.74 (1.15,2.62)** |
| **2** | **1.61 (1.10,2.37)** | **1.66 (1.11,2.50)** | 1.45 (0.93,2.25) | 1.49 (0.51,4.35) | 1.41 (0.45,4.46) | **1.64 (1.08,2.47)** |
| **3** | **1.74 (1.12,2.71)** | **1.91 (1.19,3.06)** | 1.65 (1.00,2.71) | 1.59 (0.51,4.96) | 2.08 (0.64,6.76) | **1.68 (1.04,2.73)** |
| **4** | 1.07 (0.95,1.20) | 1.06 (0.92,1.22) | 1.09 (0.94,1.26) | 0.33 (0.08,1.32) | 1.00 (0.46,2.19) | 1.07 (0.94,1.21) |
| **5** | 1.08 (0.94,1.24) | 1.08 (0.92,1.27) | 1.11 (0.94,1.31) | 0.40 (0.12,1.32) | 1.24 (0.82,1.87) | 1.06 (0.92,1.22) |
| **6** | 1.08 (0.94,1.24) | 1.07 (0.92,1.26) | 1.08 (0.94,1.26) | 1.00 (0.47,2.14) | 1.24 (0.76,2.03) | 1.07 (0.93,1.22) |
| **7** | **1.56 (1.08,2.26)** | **1.74 (1.15,2.65)** | 1.40 (0.99,1.97) | 2.83 (0.94,8.48) | 1.62 (0.75,3.51) | **1.55 (1.01,2.37)** |
| **8** | **1.34 (1.01,1.78)** | **1.48 (1.07,2.06)** | 1.23 (0.96,1.57) | 2.41 (0.75,7.75) | 1.23 (0.27,5.55) | **1.34 (1.00,1.80)** |
| **9** | **1.40 (1.03,1.89)** | **1.63 (1.12,2.36)** | 1.39 (1.00,1.92) | 0.85 (0.31,2.33) | 2.26 (0.67,7.61) | **1.35 (1.01,1.81)** |
| **10** | 1.03 (0.85,1.24) | 1.03 (0.86,1.25) | 1.07 (0.88,1.31) | 0.63 (0.32,1.24) | 0.63 (0.27,1.44) | 1.08 (0.89,1.31) |
| **11** | 0.96 (0.84,1.10) | 0.96 (0.84,1.09) | 1.01 (0.88,1.16) | 0.68 (0.41,1.12) | 0.84 (0.56,1.28) | 0.99 (0.86,1.13) |
| **12** | 0.98 (0.87,1.10) | 0.96 (0.86,1.08) | 1.01 (0.90,1.14) | 0.68 (0.42,1.11) | 0.81 (0.52,1.27) | 1.00 (0.88,1.12) |
| **13** | 1.02 (0.91,1.15) | 1.01 (0.90,1.13) | 1.05 (0.93,1.19) | 0.22 (0.04,1.12) | 0.98 (0.61,1.57) | 1.03 (0.92,1.16) |
| **14** | 1.14 (0.96,1.34) | 1.14 (0.95,1.38) | 1.12 (0.95,1.33) | 1.03 (0.59,1.79) | 1.20 (0.72,1.98) | 1.13 (0.95,1.34) |
| **15** | 1.16 (0.97,1.39) | 1.23 (1.00,1.52) | 1.16 (0.95,1.40) | 1.10 (0.61,1.98) | 1.05 (0.61,1.82) | 1.18 (0.97,1.43) |
| **16** | 1.29 (1.04,1.60) | 1.45 (1.11,1.89) | 1.27 (1.00,1.61) | 1.14 (0.62,2.07) | 1.08 (0.56,2.07) | **1.32 (1.04,1.67)** |
| **17** | 1.18 (0.97,1.42) | **1.27 (1.02,1.58)** | 1.25 (1.00,1.57) | 0.56 (0.17,1.87) | 0.78 (0.39,1.57) | 1.25 (1.00,1.55) |
| **18** | 1.10 (0.90,1.34) | 1.14 (0.93,1.39) | 1.15 (0.91,1.46) | 0.58 (0.19,1.80) | 0.80 (0.39,1.66) | 1.15 (0.92,1.42) |
| **19** | 1.04 (0.91,1.19) | 1.05 (0.91,1.22) | 1.07 (0.92,1.24) | 0.63 (0.29,1.39) | 0.61 (0.23,1.64) | 1.06 (0.92,1.22) |
| **20** | 0.99 (0.87,1.14) | 1.01 (0.88,1.16) | 1.01 (0.87,1.17) | 0.88 (0.57,1.37) | 0.75 (0.31,1.79) | 1.01 (0.87,1.15) |
| **21** | 1.02 (0.91,1.14) | 1.04 (0.92,1.17) | 1.04 (0.92,1.18) | 0.86 (0.54,1.36) | 0.64 (0.23,1.77) | 1.03 (0.92,1.16) |
| **22** | 0.97 (0.81,1.15) | 1.02 (0.86,1.22) | 0.99 (0.82,1.21) | 0.84 (0.51,1.39) | 0.78 (0.39,1.53) | 0.99 (0.82,1.18) |
| **23** | 1.07 (0.91,1.26) | 1.12 (0.93,1.35) | 1.11 (0.92,1.33) | 0.60 (0.23,1.58) | 0.79 (0.39,1.63) | 1.09 (0.91,1.30) |
| **24** | 1.08 (0.92,1.28) | 1.13 (0.93,1.37) | 1.14 (0.93,1.39) | 0.71 (0.35,1.46) | 0.89 (0.37,2.10) | 1.09 (0.91,1.30) |

Table S13: Proportions and Chi-square test ($X_{df}^{2}$) results for reported symptoms and number of GP visits in the 12 months leading up to diagnosis for cases and up to taking part in the study among CLOCS participants.

| **Characteristic** | **Cases (n = 182)** | | **Controls (n = 427)** | |  |  |
| --- | --- | --- | --- | --- | --- | --- |
|  | **N** | **%** | **N** | **%** | $\mathbf{X}_{\boldsymbol{1}}^{\boldsymbol{2}}$ | ***P*** |
| **Symptoms reported** |  |  |  |  |  |  |
| Swollen tummy | 93 | 51.1 | 0 | 0.0 | 253.6 | **<0.001** |
| Discomfort in lower tummy or pelvic area | 79 | 43.4 | 173 | 40.5 | 0.3 | 0.566 |
| Feeling constantly bloated | 72 | 39.6 | 109 | 25.5 | 11.7 | **<0.001** |
| Urge to pee more often or urgently than usual | 59 | 32.4 | 166 | 38.9 | 2.0 | 0.156 |
| Feeling tired all the time | 53 | 29.1 | 183 | 42.9 | 9.6 | **0.002** |
| Feeling full quickly | 41 | 22.5 | 51 | 11.9 | 10.3 | **0.001** |
| Constipation | 38 | 20.9 | 0 | 0.0 | 91.6 | **<0.001** |
| Loss of appetite | 31 | 17.0 | 46 | 10.8 | 3.9 | **0.046** |
| Persistent indigestion | 31 | 17.0 | 58 | 13.6 | 0.9 | 0.328 |
| Back pain | 28 | 15.4 | 188 | 44.0 | 44.5 | **<0.001** |
| Unintentional weight loss | 23 | 12.6 | 17 | 4.0 | 14.2 | **<0.001** |
| Vaginal bleeding after menopause | 14 | 7.7 | 11 | 2.6 | 7.2 | **0.007** |
| Persistent nausea | 12 | 6.6 | 20 | 4.7 | 0.6 | 0.442 |
| Pain during intercourse | 12 | 6.6 | 72 | 16.9 | 10.5 | **0.001** |
| Irregular periods | 8 | 4.4 | 88 | 20.6 | 24.1 | **<0.001** |
| None | 9 | 4.9 | 73 | 17.1 | 15.1 | **<0.001** |
|  |  |  |  |  |  |  |
| **GP visits reported** |  |  |  |  |  |  |
| 0 | 39 | 21.4 | 100 | 23.4 | 0.008* | 0.929* |
| 1 | 47 | 25.8 | 99 | 23.2 |  |  |
| 2 | 35 | 19.2 | 86 | 20.1 |  |  |
| 3 | 16 | 8.8 | 45 | 10.5 |  |  |
| 4 | 10 | 5.5 | 33 | 7.7 |  |  |
| ≥5 | 21 | 11.5 | 54 | 12.6 |  |  |
| Missing | 14 | 7.7 | 10 | 2.3 |  |  |

*Chi-square test result is for no GP visits vs. at least one GP visit.
